# Hesitação vacinal infantil e COVID-19 no Brasil: ampliando a análise a partir da percepção dos profissionais de saúde

**DOI:** 10.1590/0102-311XPT068824

**Published:** 2024-09-09

**Authors:** Tatiana Leite Muller, Fernanda Cornelius Lange, Fernando Hellmann

**Affiliations:** 1 Universidade Federal de Santa Catarina, Florianópolis, Brasil.

A hesitação vacinal é um desafio global crítico, agravado pela pandemia da COVID-19 ^1^. Recentemente, o artigo *Hesitação Vacinal Infantil e COVID-19: Uma Análise a partir da Percepção dos Profissionais de Saúde*, de Souto et al. ^2^, contribuiu significativamente ao debate desse fenômeno que tem emergido com força no contexto atual. O referido estudo explora a perspectiva dos profissionais da atenção primária à saúde (APS) no Brasil, um grupo crucial na interface com a comunidade, cujas percepções podem influenciar as decisões dos pais sobre a vacinação de seus filhos. O estudo identificou três categorias que influenciam a hesitação vacinal: o medo, a desinformação e o papel dos profissionais de saúde como mediadores de informações confiáveis. Esses *insights* são vitais para moldar estratégias de comunicação eficazes para aumentar a aceitação da vacina. Apesar da riqueza de dados e da análise detalhada apresentada por Souto et al. ^2^, acreditamos que há áreas dentro da temática da hesitação vacinal que se beneficiariam de um exame mais detalhado, ampliando a análise sobre como as percepções dos profissionais de saúde, especialmente da APS, podem ser melhor utilizadas para fortalecer as políticas de saúde pública e aumentar as taxas de vacinação infantil no Brasil e em outros contextos similares.

## Conflitos de interesse e crise de confiança na ciência

Os problemas éticos enfrentados no campo da ciência e tecnologia, particularmente nos interesses comerciais no contexto da pandemia de COVID-19, podem moldar profundamente as opiniões dos profissionais de saúde. A falta de discussão ampliada sobre o descrédito na ciência no estudo pode criar lacunas na compreensão de como as opiniões dos profissionais são formadas, destacando a necessidade de uma análise mais crítica e abrangente que contemple os conflitos de interesses na ciência e suas consequências.

No desenvolvimento e na distribuição da ciência e tecnologia, observa-se uma tendência predominante de favorecer interesses comerciais e privados, mesmo em contextos de elevado risco social, como durante pandemias ^3^. Um artigo da revista *Nature* destacou a extensão da fraude na pesquisa clínica e o impacto potencial dessas práticas nos protocolos clínicos e na saúde dos pacientes ^4^. Essas questões levantam preocupações sérias sobre a integridade e a confiabilidade dos estudos científicos. Abordar a crise de confiança na ciência e a sua crescente mercantilização é essencial para uma análise crítica mais profunda dos resultados de pesquisas, especialmente para entender como o medo se manifesta na percepção pública dos profissionais de saúde da APS.

## Agrupamento dos profissionais de saúde na análise dos dados

O artigo divulga parcialmente os resultados alcançados na pesquisa. Para enriquecer a análise dos resultados e proporcionar uma compreensão mais detalhada das dinâmicas de hesitação vacinal é fundamental que os resultados da pesquisa sejam apresentados de maneira a diferenciar claramente os papéis e as percepções dos diversos profissionais de saúde envolvidos. O agrupamento desses profissionais como um grupo homogêneo pode obscurecer nuances importantes nas percepções e práticas relacionadas à vacinação nas diferentes categorias.

Médicos, enfermeiros e agentes comunitários de saúde, por exemplo, interagem de maneiras significativamente distintas com os pacientes e suas comunidades. Ao segmentar a análise por categoria profissional, a pesquisa poderia revelar padrões específicos de hesitação vacinal e as estratégias mais eficazes para cada grupo, permitindo intervenções mais direcionadas e eficientes. Além disso, tal abordagem contribuiria para a literatura ao demonstrar como diferentes níveis de interação e responsabilidade dentro da APS influenciam as percepções e as decisões vacinais. Assim, sugere-se que futuras publicações abordem essas diferenças, para que as estratégias de comunicação e intervenção possam ser adequadamente adaptadas e implementadas.

## Ausência de discussão sobre os conselhos profissionais

O estudo faz uma crítica bem fundamentada sobre as políticas do Governo Federal durante a gestão da pandemia de COVID-19, que influenciaram negativamente na propagação de desinformação e medo, enquanto deixou de lado a influência significativa dos conselhos profissionais, que também afetam as práticas e percepções dos profissionais de saúde.

Um exemplo disso foi a pesquisa controversa do Conselho Federal de Medicina (CFM) sobre a obrigatoriedade da vacinação em crianças de 6 meses a 4 anos e 11 meses ^5^. O método adotado pelo CFM, um questionário eletrônico cujas respostas dos médicos seriam consideradas votos para futuras políticas, visava captar a percepção dos médicos brasileiros sobre a vacinação infantil. No entanto, a iniciativa recebeu severas críticas de entidades médicas e da comunidade científica por ignorar consensos científicos e os critérios técnicos do Programa Nacional de Imunizações (PNI) e por poder gerar interpretações equivocadas ^6^ ^,^ ^7^ ^,^ ^8^. A abordagem do CFM também foi vista como prejudicial à saúde pública, pois poderia reduzir a confiança nas vacinas e a adesão vacinal.

## Ampliar as recomendações do estudo

Enquanto a população enfrentava um crescente medo da vacinação, amplificado pela disseminação de desinformações, a estratégia comunicativa adotada pela APS se mostrou essencial para fortalecer a adesão aos imunizantes, particularmente no público infantil ^2^. É vital que os profissionais de saúde da APS sejam equipados com estratégias de comunicação eficazes que possam corrigir informações falsas sobre as vacinas. A conscientização e a educação continuada em saúde pública desempenham um papel crucial nesse processo, ajudando a explicar como a desinformação afeta as decisões dos profissionais de saúde e dos pais ou responsáveis. Plataformas como o Telessaúde e a Universidade Aberta do SUS (UnA-SUS) oferecem recursos valiosos para esses esforços educacionais.

Diante desses desafios, torna-se imperativo que pesquisas futuras se dediquem a desenvolver e validar estratégias que efetivamente combatam a hesitação vacinal. Essas estratégias deveriam não apenas informar, mas também engajar ativamente comunidades, promovendo uma compreensão mais profunda dos benefícios da vacinação e reforçando a confiança nas instituições de saúde.

## Considerações finais

Fundamentalmente, a hesitação vacinal representa um desafio complexo e multidimensional que necessita de uma abordagem abrangente. O estudo de Souto et al. ^2^ marca um passo importante e na direção certa, servindo como uma base sólida para futuras pesquisas e intervenções destinadas a aumentar a adesão à vacinação e a proteção de crianças e adolescentes contra a COVID-19 e outras doenças imunopreviníveis. Espera-se que o debate sobre a hesitação vacinal continue evoluindo em direção a soluções eficazes que protejam a saúde pública. É imperativo que conselhos profissionais e autoridades de saúde pública considerem esses fatores ao desenvolver políticas e programas de vacinação. Priorizar a transparência, a educação continuada e o suporte aos profissionais da APS é crucial para fortalecer seu papel como confiáveis intermediários de saúde na luta contra a hesitação vacinal.
